# Supplementary material for: Intranasal Borna Disease Virus (BoDV-1) Infection: Insights into Initial Steps and Potential Contagiosity
Source: Int J Mol Sci. 2019 Mar 15;20(6):1318. doi: 10.3390/ijms20061318 (PMC6470550; doi:10.3390/ijms20061318)
Supplement: Supplementary file 1 [file ijms-20-01318-s001.zip › TableS1.pdf]

A

cell type / structure	*p* values		
	**BoDV-1-N**	**genomic RNA**	**mRNA**
adult neurons	<0.0001	<0.0001	<0.0001
juvenile neurons	<0.0001	<0.0001	<0.0001
sustentacular cells	0.0001	-	-
globose basal cells	0.0002	-	-
horizontal basal cells	0.0016	-	-
nerve fibers	<0.0001	0.0031	0.0014
olfactory ensheathing cells	<0.0001	<0.0001	0.0016

B

cell type / structure	r_s		
	**BoDV-1-N**	**genomic RNA**	**mRNA**
adult neurons	0.7563	0.7657	0.7954
juvenile neurons	0.6813	0.7257	0.6817
sustentacular cells	0.5762	-	-
globose basal cells	0.6007	-	-
horizontal basal cells	0.5157	-	-
nerve fibers	0.6866	0.5642	0.5171
olfactory ensheathing cells	0.6866	0.705	0.5146
