# Supplementary material for: Intranasal Borna Disease Virus (BoDV-1) Infection: Insights into Initial Steps and Potential Contagiosity
Source: Int J Mol Sci. 2019 Mar 15;20(6):1318. doi: 10.3390/ijms20061318 (PMC6470550; doi:10.3390/ijms20061318)
Supplement: Supplementary file 1 [file ijms-20-01318-s001.zip › TableS3.pdf]

Primer / TaqMan® probe Sequence (5' - 3')	Concentration (nM)	GenBank® Accession-number (length / position)
*BoDV-1-N sense (p332) CGG GTA TAG GGC ATG AGA AGG ATA	200	AF158629 (96 bp / 134-229)
*BoDV-1-N antisense (p333) CAA GGC TGG GCG TTA CTG TAT G	300	
BoDV-1-N probe CGC CGT GAC TGG TCT AAC AAT GCC ACT	200	
GAPDH sense (p338) GGT CTA CAT GTT CCA GTA TGA CTC T	100	NM017008 (82 bp / 972-1053)
*GAPDH antisense (p339) GTT GAT GAC CAG CTT CCC ATT CT	100	
GAPDH probe CGG CAA GTT CAA CGG CAC AGT CAA GGC	100	
