# Supplementary material for: Intranasal Borna Disease Virus (BoDV-1) Infection: Insights into Initial Steps and Potential Contagiosity
Source: Int J Mol Sci. 2019 Mar 15;20(6):1318. doi: 10.3390/ijms20061318 (PMC6470550; doi:10.3390/ijms20061318)
Supplement: Supplementary file 1 [file ijms-20-01318-s001.zip › TableS2.pdf]

A

Immunohistochemistry BoDV-1-N

		Time post infection	3 hpi	18 hpi	1 dpi	2 dpi	4 dpi	7 dpi	14 dpi	21 dpi
**Adult neurons**	Score	0	5	5	5	5	3	3	0	0
		1	0	0	0	0	2	2	0	0
		2	0	0	0	0	0	0	2	0
		3	0	0	0	0	0	0	3	2
		Arithmetic mean	0	0	0	0	0.4	0.4	2.6	3
**Juvenile neurons**	Score	0	5	5	5	5	4	5	0	0
		1	0	0	0	0	1	0	2	0
		2	0	0	0	0	0	0	1	1
		3	0	0	0	0	0	0	2	1
		Arithmetic mean	0	0	0	0	0.2	0	2	2.5
**Sustentacular cells**	Score	0	5	5	5	5	5	5	2	0
		1	0	0	0	0	0	0	1	0
		2	0	0	0	0	0	0	2	1
		3	0	0	0	0	0	0	0	1
		Arithmetic mean	0	0	0	0	0	0	1	2.5
**Globose basal cells**	Score	0	5	5	5	5	5	4	2	0
		1	0	0	0	0	0	1	1	0
		2	0	0	0	0	0	0	2	1
		3	0	0	0	0	0	0	0	1
		Arithmetic mean	0	0	0	0	0	0.2	1	2.5
**Horizontal basal cells**	Score	0	5	5	5	5	5	5	3	0
		1	0	0	0	0	0	0	0	1
		2	0	0	0	0	0	0	2	1
		3	0	0	0	0	0	0	0	0
		Arithmetic mean	0	0	0	0	0	0	0.8	1.5
**Nerve fibers**	Score	0	5	5	5	5	5	5	0	0
		1	0	0	0	0	0	0	1	0
		2	0	0	0	0	0	0	4	0
		3	0	0	0	0	0	0	0	2
		Arithmetic mean	0	0	0	0	0	0	1.8	3
**Olfactory ensheathing cells**	Score	0	5	5	5	5	5	5	0	0
		1	0	0	0	0	0	0	1	0
		2	0	0	0	0	0	0	4	0
		3	0	0	0	0	0	0	0	2
		Arithmetic mean	0	0	0	0	0	0	1.8	3

B

***In situ* hybridization viral genomic RNA (gRNA)**

		Time post infection	3 hpi	18 hpi	1 dpi	2 dpi	4 dpi	7 dpi	14 dpi	21 dpi
**Adult neurons**	Score	0	5	5	5	5	5	2	0	0
		1	0	0	0	0	0	2	0	0
		2	0	0	0	0	0	1	5	2
		3	0	0	0	0	0	0	0	0
		Arithmetic mean	0	0	0	0	0	0.8	2	2
**Juvenile neurons**	Score	0	5	5	5	5	5	3	0	0
		1	0	0	0	0	0	1	5	1
		2	0	0	0	0	0	1	0	1
		3	0	0	0	0	0	0	0	0
		Arithmetic mean	0	0	0	0	0	0.6	1	1.5
**Sustentacular cells**	Score	0	5	5	5	5	5	5	5	2
		1	0	0	0	0	0	0	0	0
		2	0	0	0	0	0	0	0	0
		3	0	0	0	0	0	0	0	0
		Arithmetic mean	0	0	0	0	0	0	0	0
**Globose basal cells**	Score	0	5	5	5	5	5	5	5	2
		1	0	0	0	0	0	0	0	0
		2	0	0	0	0	0	0	0	0
		3	0	0	0	0	0	0	0	0
		Arithmetic mean	0	0	0	0	0	0	0	0
**Horizontal basal cells**	Score	0	5	5	5	5	5	5	5	2
		1	0	0	0	0	0	0	0	0
		2	0	0	0	0	0	0	0	0
		3	0	0	0	0	0	0	0	0
		Arithmetic mean	0	0	0	0	0	0	0	0
**Nerve fibers**	Score	0	5	5	5	5	5	3	3	0
		1	0	0	0	0	0	2	2	1
		2	0	0	0	0	0	0	0	1
		3	0	0	0	0	0	0	0	0
		Arithmetic mean	0	0	0	0	0	0.4	0.4	1.5
**Olfactory ensheathing cells**	Score	0	5	5	5	5	5	4	0	0
		1	0	0	0	0	0	1	2	1
		2	0	0	0	0	0	0	3	1
		3	0	0	0	0	0	0	0	0
		Arithmetic mean	0	0	0	0	0	0.2	1.6	1.5

C

***In situ* hybridization messenger RNA (mRNA)**

		Time post infection	3 hpi	18 hpi	1 dpi	2 dpi	4 dpi	7 dpi	14 dpi	21 dpi
**Adult neurons**	Score	0	5	5	5	5	3	1	0	0
		1	0	0	0	0	0	3	1	0
		2	0	0	0	0	2	1	3	1
		3	0	0	0	0	0	0	1	1
		Arithmetic mean	0	0	0	0	0.8	1	2	2.5
**Juvenile neurons**	Score	0	5	5	5	5	5	5	0	0
		1	0	0	0	0	0	0	3	1
		2	0	0	0	0	0	0	1	0
		3	0	0	0	0	0	0	1	1
		Arithmetic mean	0	0	0	0	0	0	1.6	2
**Sustentacular cells**	Score	0	5	5	5	5	5	5	5	2
		1	0	0	0	0	0	0	0	0
		2	0	0	0	0	0	0	0	0
		3	0	0	0	0	0	0	0	0
		Arithmetic mean	0	0	0	0	0	0	0	0
**Globose basal cells**	Score	0	5	5	5	5	5	5	5	2
		1	0	0	0	0	0	0	0	0
		2	0	0	0	0	0	0	0	0
		3	0	0	0	0	0	0	0	0
		Arithmetic mean	0	0	0	0	0	0	0	0
**Horizontal basal cells**	Score	0	5	5	5	5	5	5	5	2
		1	0	0	0	0	0	0	0	0
		2	0	0	0	0	0	0	0	0
		3	0	0	0	0	0	0	0	0
		Arithmetic mean	0	0	0	0	0	0	0	0
**Nerve fibers**	Score	0	5	5	5	5	5	5	3	0
		1	0	0	0	0	0	0	1	1
		2	0	0	0	0	0	0	1	1
		3	0	0	0	0	0	0	0	0
		Arithmetic mean	0	0	0	0	0	0	0.6	1.5
**Olfactory ensheathing cells**	Score	0	5	5	5	5	5	5	3	0
		1	0	0	0	0	0	0	0	1
		2	0	0	0	0	0	0	1	1
		3	0	0	0	0	0	0	1	0
		Arithmetic mean	0	0	0	0	0	0	1	1.5
