# Supplementary figures and images for: Intranasal Borna Disease Virus (BoDV-1) Infection: Insights into Initial Steps and Potential Contagiosity
Source: Int J Mol Sci. 2019 Mar 15;20(6):1318. doi: 10.3390/ijms20061318 (PMC6470550; doi:10.3390/ijms20061318)

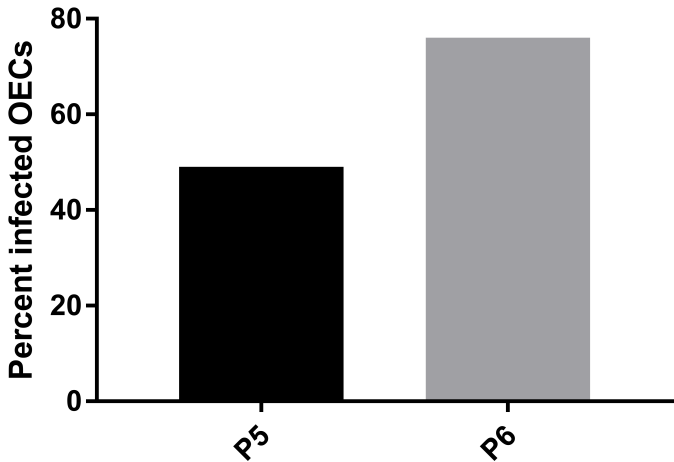

Supplement: Supplementary file 1 [file ijms-20-01318-s001.zip › FigureS2.pdf]

**A**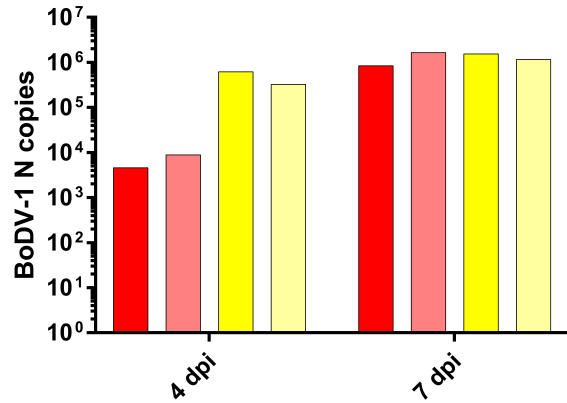

OM gRNA  
OM mRNA  
OEC gRNA  
OEC mRNA

**B**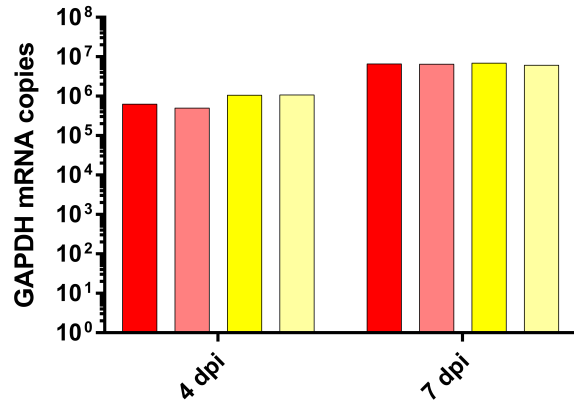

OM gRNA  
OM mRNA  
OEC gRNA  
OEC mRNA

Supplement: Supplementary file 1 [file ijms-20-01318-s001.zip › FigureS1.pdf]
